# Comparative Genomic Analysis of a Panton–Valentine Leukocidin-Positive ST22 Community-Acquired Methicillin-Resistant *Staphylococcus aureus* from Pakistan

**DOI:** 10.3390/antibiotics11040496

**Published:** 2022-04-08

**Authors:** Nimat Ullah, Samavi Nasir, Zaara Ishaq, Farha Anwer, Tanzeela Raza, Moazur Rahman, Abdulrahman Alshammari, Metab Alharbi, Taeok Bae, Abdur Rahman, Amjad Ali

**Affiliations:** 1 Atta-ur-Rahman School of Applied Biosciences (ASAB), National University of Sciences and Technology (NUST), Sector H-12, Islamabad 44000, Pakistan; nullah.phdabs12asab@asab.nust.edu.pk (N.U.); snasir.msib08asab@student.nust.edu.pk (S.N.); zishaq.msib07asab@student.nust.edu.pk (Z.I.); fanwar.phdabs17asab@student.nust.edu.pk (F.A.); tanzeela.raza12@gmail.com (T.R.); a.rahman@asab.nust.edu.pk (A.R.); 2 Department of Microbiology and Immunology, Indiana University School of Medicine-Northwest, 3400 Broadway, Gary, IN 46408, USA; tbae@iun.edu; 3 School of Biological Sciences, University of the Punjab, Quaid-i-Azam Campus, Lahore 54590, Pakistan; moaz.sbs@pu.edu.pk; 4 Department of Pharmacology and Toxicology, College of Pharmacy, King Saud University, P.O. Box 2455, Riyadh 11451, Saudi Arabia; mesalharbi@ksu.edu.sa

**Keywords:** comparative genomic analysis, CA-MRSA, EMRSA-15, PVL-positive, ST22

## Abstract

*Staphylococcus aureus* (*S. aureus*) ST22 is considered a clinically important clone because an epidemic strain EMRSA-15 belongs to ST22, and several outbreaks of this clone have been documented worldwide. We performed genomic analysis of an *S. aureus* strain Lr2 ST22 from Pakistan and determined comparative analysis with other ST22 strains. The genomic data show that Lr2 belongs to *spa*-type t2986 and harbors staphylococcal cassette chromosome *mec* (SCC*mec*) type IVa(2B), one complete plasmid, and seven prophages or prophage-like elements. The strain harbors several prophage-associated virulence factors, including Panton–Valentine leukocidin (PVL) and toxic shock syndrome toxin (TSST). The single nucleotide polymorphism (SNPs)-based phylogenetic relationship inferred from whole genome and core genome revealed that strain Lr2 exhibits the nearest identities to a South African community-acquired methicillin-resistant *S. aureus* (CA-MRSA) ST22 strain and makes a separate clade with an Indian CA-MRSA ST22 strain. Although most ST22 strains carry *blaZ*, *mecA*, and mutations in *gyrA*, the Lr2 strain does not have the *blaZ* gene but, unlike other ST22 strains, carries the antibiotic resistance genes *erm(C)* and *aac(6′)-Ie-aph(2″)-Ia*. Among ST22 strains analyzed, only the strain Lr2 possesses both PVL and TSST genes. The functional annotation of genes unique to Lr2 revealed that mobilome is the third-largest Cluster of Orthologous Genes (COGs) category, which encodes genes associated with prophages and transposons. This possibly makes methicillin-resistant *S. aureus* (MRSA) Lr2 ST22 strain highly virulent, and this study would improve the knowledge of MRSA ST22 strains in Pakistan. However, further studies are needed on a large collection of MRSA to comprehend the genomic epidemiology and evolution of this clone in Pakistan.

## 1. Introduction

*Staphylococcus aureus* (*S. aureus*) has long been considered an important human pathogen that causes both hospital- and community-acquired (HA and CA) infections. This pathogen is also well-known for acquiring resistance to a variety of antibiotics [1,2]. Particularly, methicillin resistance is becoming more common in *S. aureus*, posing a growing public health threat with substantial mortality and morbidity. The methicillin-resistant *S. aureus* (MRSA) produces a low-affinity penicillin-binding protein (PBP2a), which enables it to confer resistance to almost all beta-lactam antibiotics [3,4]. The PBP2a is encoded by the gene *mecA*, which is carried by a large mobile genetic element known as staphylococcal cassette chromosome *mec* (SCC*mec*) [5]. In the past, MRSA caused mostly hospital-acquired infections; however, now, it also causes community-acquired infections, including necrotizing pneumonia and skin and soft tissue infection (SSTI) [6]. Apart from humans, MRSA can also colonize other animal species, particularly livestock, and CA-MRSA infections can also be caused by livestock-associated MRSA (LA-MRSA) [7].

Compared with HA-MRSA, the CA-MRSA strains belong to a diverse lineage with smaller SCC*mec*, for example, SCC*mec* IV, and harbors distinct virulence factors, particularly Panton–Valentine leukocidin (PVL) [8]. PVL is a two-component toxin encoded by prophage-associated genes *lukF-PV* and *lukS-PV* [9,10]. The toxin targets phagocytic leucocytes and triggers leukocyte lysis and/or apoptosis by forming pores. Therefore, PVL-positive CA-MRSA strains cause various infections, particularly necrotizing pneumonia and SSTI [11]. Although not all CA-MRSA contain the *pvl* gene, it can be considered as a molecular marker for CA-MRSA along with the SCC*mec* type IV because PVL is associated with the global spread of CA-MRSA [12]. The well-known clones of PVL-positive CA-MRSA include ST8, frequently found in the United States [13]; ST80, a Europe clone [10]; ST59, reported in Taiwan [14]; ST30, a global strain [15]; and ST22 (SCC*mec* IV/PVL-positive), which emerged in the United Kingdom and is now spreading worldwide [16]. *S. aureus* ST22 is considered a clinically important clone because an epidemic strain EMRSA-15 belongs to ST22. The EMRSA-15 emerged in the United Kingdom in the 1990s [17] and very quickly expanded all over Europe, Australia, and Asia [18]. EMRSA-15 has SCC*mec* type IV, a deletion of 2268 bp region in fibronectin-binding protein (FnBP) locus, and a point mutation in the *ureC* gene, and it is resistant to fluoroquinolone and macrolide. The genomic analysis of ST22 strains revealed that the epidemic clone EMRSA-15 makes a distinct clade (ST22-A clade) [18].

Although there is a wealth of literature available on comprehensive epidemiological and molecular characterization of MRSA in the USA, Europe, and Asia, there is a scarcity of detailed genomic characterization of MRSA strains in Pakistan. According to some recent studies, Pakistan has a greater rate of MRSA infection, and the data suggest that MRSA clones are becoming more diverse [19,20,21]. The whole-genome analysis provides high resolution in both global and local outbreak investigations as well as further exploring genes associated with pathogenicity and antibiotic resistance [22]. Therefore, whole-genome-based approaches have become an indispensable tool for the real-time comparative genomic study of a variety of pathogens in terms of antibiotic resistance, the emergence of new virulent clones, and niche adaptation [23]. Furthermore, investigating virulence- and antibiotic resistance-associated genes can help identify risk factors for MRSA infection and develop efficient infection control programs. Given the lack of genome-based surveillance and epidemiological studies of MRSA in Pakistan, we aimed to study a CA-MRSA ST22 strain from Pakistan at the genomic level and compare the virulence and antibiotic resistance genes and evolutionary relationships to other ST22 strains. To our knowledge, this is the first genome-based analysis of PVL-positive CA-MRSA ST22 from Pakistan, and we found many genomic differences compared to other complete genomes of MRSA ST22 strains.

## 2. Results

### 2.1. Preliminary Identification and Phenotypic Antibiotic Resistance Pattern 

The isolate Lr2 was found positive for coagulase and catalase while negative for oxidase. The isolate was resistant to oxacillin, methicillin, ampicillin, erythromycin, gentamicin, streptomycin, clindamycin, ciprofloxacin, linezolid, and tetracycline but susceptible to vancomycin, chloramphenicol, and rifampicin (Table 1). The amplification of the 500 bp *mecA* gene confirmed the nature of methicillin resistance.

### 2.2. Genomic Features and Epidemiological Typing

The genomic DNA (gDNA) was successfully sequenced, and a total of 1,363,009 reads with a mean length of 565 bp (272-fold sequence coverage) were obtained. The kmerFinder 2.0 identified the isolate as *S. aureus*. The N_50_, N_75_, and L_50_ values are 131,746, 94,139, and 7, respectively; de novo assembly resulted in 52 contigs (>500 bp), and the size of the largest contig is 425,597 bp. The genome size of Lr2 is 2,831,239 bp with 32.7% GC content. Genome annotation predicted 2835 total genes, of which 2768 are CDSs. The number of predicted tRNA are 56 and rRNA are 7 (5S = 4, 16S = 2, 23S = 1). In silico genome-based epidemiological typing revealed that strain Lr2 belongs to ST22, *spa*-type t2986, and carries SCC*mec* type IVa(2B) (Table 2).

### 2.3. Predicted Antibiotic Resistance Determinants and Virulence Factors

**Figure 1:**
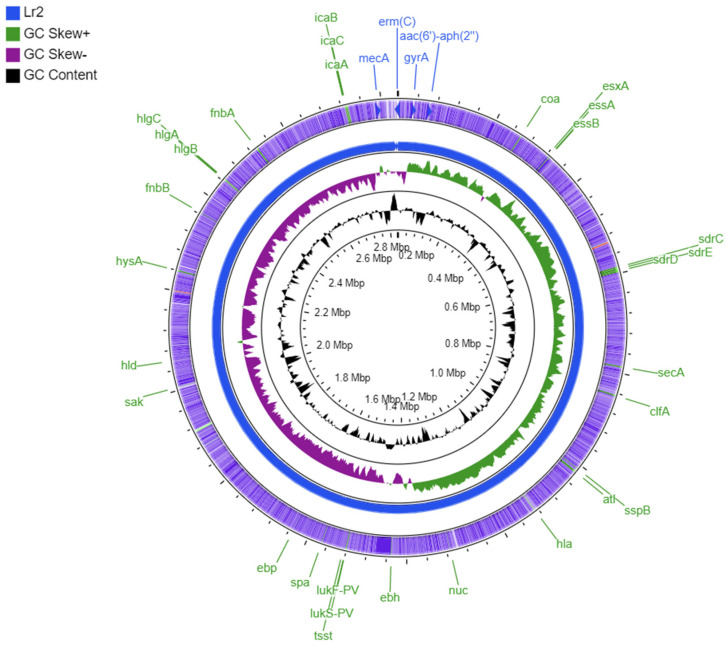
Circular visualization of Lr2 genome via CGViewer, showing virulence factors (light green) and antibiotic resistance determinants (light blue).

The Lr2 genome contains a methicillin resistance gene *mecA*, an aminoglycoside resistance gene *aac*(*6′*)*-Ie-aph*(*2″*)*-Ia*, a point mutation in *gyrA* conferring resistance to fluoroquinolones, and *erm(C)*—a plasmid-associated gene conferring resistance to lincosamide, macrolide (erythromycin), and streptogramin. However, the *blaZ* gene conferring penicillin resistance was found absent in Lr2 (Table 1 and Figure 1).

The Virulence Factor Database (VFDB) predicated 62 virulence factors responsible for adherence, protein secretion, immune evasion, and toxin production. Adherence-associated genes are autolysin (*atl*); clumping factor A (*clfA*) and B (*clfB*); an elastin binding protein (*ebp*); cell-wall-associated fibronectin-binding protein (*ebh*); collagen adhesion (*cna*); fibrinogen-binding protein (*efb*); fibronectin-binding protein A (*fnbA*) and B (*fnbB*); Ser-Asp rich fibrinogen-binding protein C (*sdrC*), D (*sdrD*), and E (*sdrE*); intercellular adhesin (*icaA*, *icaB*, and *icaC*); and staphylococcal protein A (*spa*) (Figure 1). The virulome also consists of several exoenzymes genes, including hyaluronate lyase (*hysA*), staphylokinase (*sak*), cysteine protease (*sspB* and *sspC*), serine V8 protease (*sspa*), lipase (*geh* and *lip*), staphylocoagulase (*coa*), and thermonuclease (*nuc*). However, a six gene-cluster (*splA*, *splB*, *splC*, *splD*, *splE*, and *splF*) of serine was absent in Lr2 (Figure 1). The Lr2 genome also contains virulence factors associated with the host immune evasion, such as staphylococcal complement inhibitor (*scn*), IgG-binding protein (*sbi*), and chemotaxis inhibiting protein (*chp*) (Figure 1). It also carries type VII secretion system associated virulence factors, including soluble cytosolic (*esaB* and *esaG*) and membrane-associated protein A (*essA*), B (*essB*), and C (*essC*), and A (*esxA*) but no *esxB*, *esxC*, *esxD*, *esaD*, and *esaE* were found in Lr2 genome (Figure 1).

The genome of Lr2 carries toxin associated genes, particularly alpha-hemolysin gene (*hla*), gamma hemolysin (*hlgA*, *hlgB*, and *hlgC*), delta hemolysin gene (*hld*), exotoxins (*set10*, *set13*, *set15*, and *set16*), enterotoxin-like O (*selo*), exotoxins (*set1*, *set2*, *set4*, *set7*, *set10*, *set13*, *set15*, and *set16*), enterotoxin-like M (*selm*), enterotoxin-like N (*seln*), enterotoxin B (*seg*), PVL (*lukF-PV* and *lukS-PV*), and TSST (tsst-1). However, beta-hemolysin gene (*hlb*), exfoliative toxin type A (*eta*), enterotoxin A (*sea*), enterotoxin Yent1 (*yent1*), enterotoxin-like K (*selk*), exotoxins (*set34*, *set37*, and *set39*), and leukotoxins (*lukM*, *lukD*, and *lukE*) were found absent (Figure 1).

### 2.4. Predicted Plasmids and Prophages

PlasmidFinder identified one plasmid of 2402 bp length with 99.75% sequence similarity to *S. aureus* strain E14 plasmid pDLK1 (GU562624.1). The pDLK1 plasmid consists of an erythromycin resistance gene (*emrC*) and a replication gene (*repL*), and it encodes no other factors. 

The genome of Lr2 strain presents seven putative prophages including a complete prophage (PHAGE_Staphy_tp310_3), five incomplete prophages (PHAGE_Bacill_IEBH, PHAGE_Staphy_PT1028, PHAGE_Clostr_phiC2, PHAGE_Staphy_phiPVL_CN125, and PHAGE_Staphy_96), and a questionable prophage (PHAGE_Staphy_phiN315) (Table 3). Several virulence factors were predicted in the identified prophages, e.g., *sec*, *sell*, and *tsst* in the prophage PHAGE_Bacill_IEBH; the *ebp*, *lukF-PV*, and *lukS-PV* in PHAGE_Staphy_phiPVL_CN125; the *seg*, *sei*, *yent2*, *selm*, *seln*, and *selo* in PHAGE_Staphy_96; *sak*, *chp*, and *scn* in PHAGE_Staphy_tp310_3; and *cna* in PHAGE_Staphy_phiN315 (Table 3). However, no antibiotic resistance gene was found in any of the identified prophages.

### 2.5. Comparative Phylogenetic Analysis 

**Figure 2:**
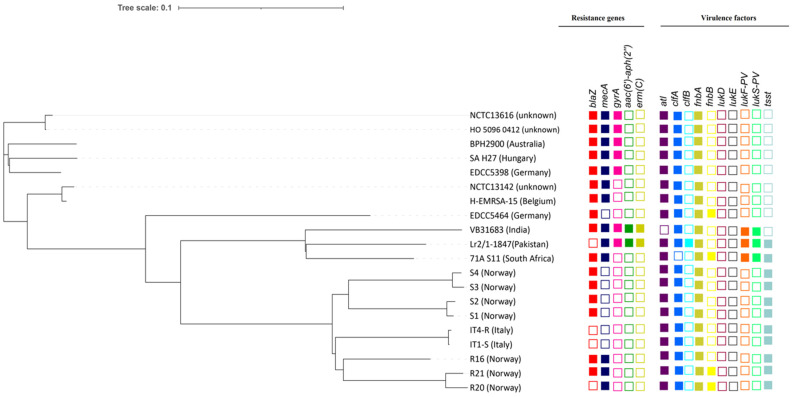
Whole-genome-based SNPs phylogenetic tree of CA-MRSA ST22 strain Lr2 and other ST22 strains. The heatmap shows presence and absence of antimicrobial resistance determinants and virulence factors.

The whole-genome-based single nucleotide polymorphisms (SNPs) phylogenetic analysis of the Lr2 ST22 strain with all other ST22 strains indicated that the Lr2 strain exhibits the nearest identities to South African CA-MRSA ST22 strain 71A_S11 (CP010940) and Indian strain CA-MRSA ST22 VB31683 (CP035671) (Figure 2).

### 2.6. Comparative Analysis of Antibiotic Resistance Determinants and Virulence Factors

The heatmap shows that aminoglycoside resistance gene *aac*(*6′*)*-Ie-aph*(*2″*)*-Ia* and a plasmid-associated gene *erm(C)* conferring resistance to lincosamide, macrolide (erythromycin), and streptogramin are only present in Lr2 and Indian strain VB31683. Both Lr2 and Indian strain VB31683 also have a point mutation in *gyrA*, conferring fluoroquinolones resistance, which is absent in a majority of ST22 strains. However, the penicillin-resistance gene (*blaZ*) is absent in Lr2, while it is present in almost all ST22 strains. In contrast, the South African strain 71A_S11, a sister strain to Lr2, carries only *blaZ* and *mecA* genes (Figure 2). However, South African strain 71A_S11 has a very similar profile of virulence factors to that of the Lr2 strain since both TSST (tsst) and PVL (*lukF-PV* and *lukS-PV*) genes are only present in these two ST22 strains. In addition, Lr2 strains also harbor clumping factor B (*clfB*), which is absent in South African strain 71A_S11 and all other ST22 strains. Interestingly, leukotoxin D (*lukD*) and E (*lukE*) were absent in all ST22 strains, including the Lr2 strain (Figure 2).

### 2.7. Pan-Genome Analysis and Functional Annotation 

**Figure 3:**
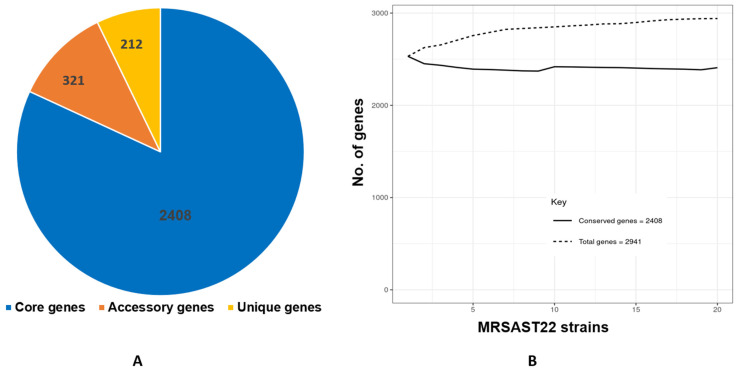
The pan genome of MRSA ST22 strains. (**A**) The pie chart shows number of genes in core, accessory, and unique genomes of twenty genomes of ST22. (**B**) The pan genome vs. core genome plot of ST22 strains.

The pan genome of MRSA ST22 strains consists of 2941 genes, of which 2408 (81.8%) genes are part of the core genome, 321 (10.9%) genes are accessory, and 212 (7.2%) genes are unique (Figure 3A). The plot of the pan and core genome shows fluctuation in the number of pan and core genes, which suggests that pan- and core-genome sizes are not stable (Figure 3B).

**Figure 4:**
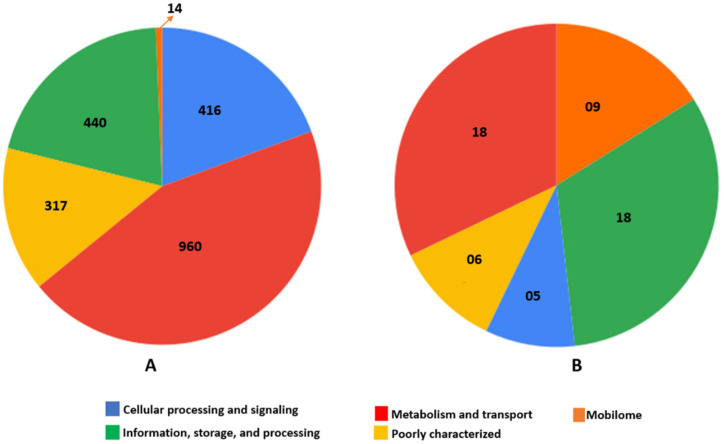
Functional annotation of core genome and genes unique to strain Lr2. (**A**) Distribution of COGs in core genome of MRSA strains ST22. (**B**) Distribution of COGs unique to strain Lr2.

The functional annotation of core genes shows that 960 (44.7%) Cluster of Orthologous Genes (COGs) are involved in metabolism and transport; 440 (20.5%) in information, storage, and processing; and 416 (19.4%) in cellular processing and signaling; 317 (14.8%) are poorly characterized, and 14 (0.7%) are found to be associated with mobilome (Figure 4A). The functional annotation of the genes unique to MRSA strain Lr2 ST22 revealed that 18 (32.1%) COGs are involved in metabolism and transport, 18 (32.1%) in information, storage, and processing, 9 (16.1%) are associated with mobilome, 6 (10.7%) are poorly characterized (either involve in general function and/or unknown function), and 5 (8.9%) are involved in cellular processing and signaling (Figure 4B).

**Figure 5:**
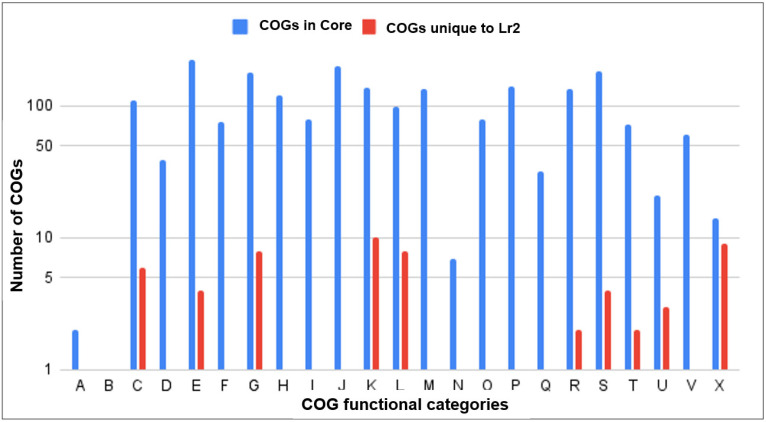
Comparative functional categories of COG in core genome of ST22 strains and COGs unique to strain Lr2.

The largest core-genome category of MRSA strains ST22 consists of genes with functions associated with metabolism and transport, which are further categorized as C (110 genes), involved in energy conversion and production; E (223 genes), involved in transport and metabolism of amino acid; F (75 genes), involved in transport and metabolism of nucleic acids; G (179 genes), involved in transport and metabolism of carbohydrates; H (121 genes), involved in transport and metabolism of coenzyme; I (80 genes), involved in lipid transport and metabolism; P (140 genes), involved in inorganic ions transport and metabolism; and Q (32 genes), involved in biosynthesis, transport, and catabolism of secondary metabolites (Figure 5). The second-largest COGs category with functions related to information, storage, and processing are individually categorized as A (2 genes), involved in modification and processing of RNAs; B (1 gene), involved in structure and dynamics of chromatins; J (200 genes), involved in ribosomal structure, biogenesis, and translation; K (139 genes), involved in transcription; and L (98 genes), involved in repair, recombination, and replication (Figure 5). The COGs category of core genome containing the lowest number of genes belonging to mobilome, prophages, and transposons are categorized as X, which accounts for about 0.7% of the total core genome (Figure 5).

As expected, the largest category of COGs unique to Lr2 involved in metabolism and transport and are individually categorized as six genes in the C category (involved in energy conversion and production), four in the E category (involved in transport and metabolism of amino acid), and eight in the G category (involved in transport and metabolism of carbohydrates). No protein of F (involved in transport and metabolism of nucleic acids), H (involved in transport and metabolism of coenzyme), I (involved in lipid transport and metabolism), P (involved in inorganic ions transport and metabolism), and Q (involved in biosynthesis, transport, and catabolism of secondary metabolites) category was found (Figure 5). Interestingly, the third-largest category of COGs unique to Lr2 belongs to the X category, which encodes genes associated with prophages and transposons (Figure 5). Cellular processing and signaling is the lowest COGs category with five genes, which are individually classified as three genes in the U category (involved in intracellular trafficking, secretions, and vesicular transport) and two in the T category (involved in signal transduction mechanisms) (Figure 5).

In the pan genome of MRSA ST22 strains, 5 COGs were found to be present in all 19 strains while absent in the Lr2 strain. Functional annotation showed that those COGs are involved in transposase (COG3666) and putative lipase (COG5153), which is essential for the disintegration of autophagic bodies inside the vacuole.

**Figure 6:**
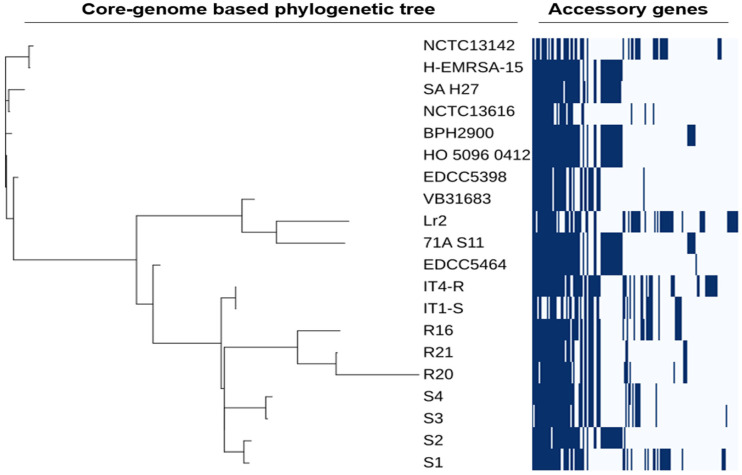
Core-genome SNPs-based tree and heatmap of presence and absence of accessory genes in 20 strains of MRSA ST22.

The core-genome phylogenetic analysis grouped the Lr2 strain with a South African ST22 strain 71A S11 strain and an Indian ST22 strain VB31683 (Figure 6). The phylogenetic relationships determined from the core genome are in agreement with the phylogenetic tree from whole-genome-based SNPs (Figure 2). The strains share more than 80% of genes; however, the accessory genome of MRSA strain Lr2 contains many genes that are absent in most of the strains (Figure 6).

## 3. Discussion

The MRSA SCC*mec* type IV ST22 is a clinically important clone (EMRSA-15), and several outbreaks of ST22 CA-MRSA and/or CA-MSSA have been documented worldwide [24,25,26]. The ST22 clone is considered a genotype of HA-EMRSA-15; however, the presence of ST22 clones in the general population and its correlation with community settings have been reported recently [27]. The dissemination of MRSA from hospitals to the community and vice versa and the emergence of strains resistant to β-lactams is a major cause of concern worldwide. Consequently, tracking of emerging clones of MRSA at the genomic level is required to prevent further spread and to guide the development of rapid diagnostic tools and therapy. Although a small number of studies from Pakistan molecularly characterized MRSA, including SCC*mec*, PFGE, and MLST typing, as well as some studies that investigated PVL genes [19,28,29,30], none of them performed a genome-based analysis. This shows that there are limited epidemiological and genomic data on MRSA reported from Pakistan, leaving a substantial knowledge gap in our understanding of this important human pathogen that what are the dominant clones of MRSA circulating in Pakistan and what makes them antibiotic-resistant and virulent. Therefore, we performed an in-depth genome-based analysis of a CA-MRSA ST22 strain from Pakistan and its comparative genomic analysis with other MRSA ST22 available in the NCBI database.

Genome analysis indicated that Lr2 belongs to MLST type ST22, spa-type t2986, and SCC*mec* type IVa(2B). According to previous studies, *SCCmec* types IV is associated with CA-MRSA, whereas HA-MRSA mostly exhibits *SCCmec* types I, II, or III [22,31]. The strain was also found to be positive for PVL (*lukF-PV* and *lukS-PV*), which is commonly considered a CA-MRSA marker [22,32]. Another study reported a high rate of MRSA PVL-positive isolates from Pakistan, which were classified as community-associated [28]. The PVL-positive strains are responsible for abscesses formation, tissue necrosis, and increased inflammatory responses [33]. In addition to PVL, TSST-producing MRSA strains tend to cause more complex infections. The presence of both *tsst* and PVL (*lukF-PV* and *lukS-PV*) genes poses a concern for increased virulence of Lr2. A recent study also reported both PVL (*lukF-PV* and *lukS-PV*) genes and the *tsst* gene in MRSA isolates from this region [19]; however, the presence of PVL genes in combination with *tsst* gene appears to be extremely rare. The *tsst* and PVL (*lukF-PV* and *lukS-PV*) genes were found on PHAGE_Staphy_PT1028 and PHAGE_Staphy_phiPVL_CN125, respectively. The genes encoding staphylococcal enterotoxins (i.e., *selm*, *seg*, *yent2*, *sei*, *selo*, and *seln*) were found on PHAGE_Staphy_96, which are involved in staphylococcal food poisoning and belong to the enterotoxin gene cluster (*egc*) [34]. In addition, the staphylococcal complement inhibitor (SCIN) encoding gene *scn* as well as chemotaxis inhibiting protein of staphylococcus (CHIPS) encoding gene *chp* were also found on prophages of the sequenced strain. These proteins have a significant role in the host-pathogen interaction and help the pathogen to evade the host’s immune response [35]. Therefore, the prophages predicted in this strain can act as a reservoir of virulence factors and could contribute to strain evolution towards high virulency and pose a serious threat [36]. Aside from the prophage-associated virulence factors, genes involved in the *S. aureus* type VII secretion system, such as *essA*, *essB*, *essC, esxA, esaA*, *esaB*, and *esaG*, were also present in PVL-positive CA-MRSA strain Lr2 (Figure 1), which promote bacterial persistence [37]. The *esxA* gene is involved in the colonization and dissemination of *S. aureus* as well as triggering T-cell immune response [38]. The genes encoding adhesion factors, including fibrinogen-binding protein (*efb*), collagen-binding protein (*cna*), Ser-Asp rich fibrinogen-binding proteins C (*sdrC*), D (*sdrD*), and E (*sdrE*), elastin-binding protein (*ebp*), clumping factors A (*clfA*), and fibronectin-binding protein (*fnbA*) and (*fnbB*), were also found in the studied genome. These surface components have several functions, including adhesion to host cells, evasion of immunological responses, and biofilm formation [39].

In silico screening of antibiotic-resistant determinants shows that *mecA* gene, *aac*(*6′*)*-Ie-aph*(*2″*)*-Ia*, and a *gyrA* gene are present on the Lr2 chromosome. However, *erm(C)*, which confers resistance to lincosamide, macrolide (erythromycin), and streptogramin was found on plasmid pDLK1 (Figure 1). It is suggested that *erm(C)* is essential to this isolate for environmental adaptation [40]. It was noticed that *blaZ* gene is absent in the studied genome, and since the *blaZ* gene (β-lactamase) is carried by a transposon Tn*552* located on a large plasmid [41], it was probably eliminated due to the curing of that plasmid.

The SNPs phylogenetic relationship inferred from whole genome and core genome revealed that strain Lr2 exhibits the nearest identities to South African CA-MRSA ST22 strain 71A_S11 (CP010940) and makes a separate clade with Indian strain CA-MRSA ST22 VB31683 (CP035671) (Figure 2). The heatmap of accessory genes comparison revealed variation in accessory genes among different strains showing gain or loss of genes as well as mobile genetic elements (MGEs) such as plasmids, prophages, and *SCCmec* element, etc. (Figure 6). This pattern of variation in genes was also observed in previous studies [22,42]. These variations can be attributed to their distinct genetic makeup, as we noticed diverse distribution of antibiotic resistance determinants and virulence factors in these strains. We observed that *blaZ*, *mecA*, and *gyrA* antibiotic resistance genes are present in most ST22 strains. However, *blaZ* was absent in strains Lr2, R20, IT1-S, and IT4-R, while *aac*(*6′*)*-Ie-aph*(*2″*)*-Ia* and *erm(C)* are unique to Lr2 and Indian strain VB31683. A similar pattern of variation in virulence factors was also observed as the PVL genes (*lukF-PV* and *lukS-PV*) were only found in Lr2, VB31683, and 71A_S11 strains. A virulence factor clumping factor B (*clfB*), was only present in Lr2 (Figure 2). These additional genes associated with antibiotic resistance and virulence are expected to make Lr2 more resistant to antibiotics and virulent.

The pan-genome analysis of MRSA ST22 strains revealed that more than 80% of genes are part of the core genome, which shows high conservancy in these strains. The core genome annotation using the Cluster of Orthologous Gene (COG) database revealed the two largest core genome categories with functions associated with metabolism and transport as well as related to information, storage, and processing. Previous studies also reported high conservancy in the *S. aureus* core genome and that the core genes are mostly associated with metabolism, replication, and information storage and processing [22,43,44]. However, genes belonging to mobilome, prophages, and transposons showed the lowest proportion. Interestingly, mobilome is the third-largest category of COGs unique to strain Lr2 (Figure 4). This abundance of mobile genetic elements in the Lr2 strain likely contributes to its increased virulence and antibiotic resistance.

## 4. Materials and Methods

### 4.1. Isolation and Antibiotic Susceptibility Testing

*S. aureus* isolates were collected from hospitals in Peshawar, Rawalpindi/Islamabad, and Lahore, and 4 isolates were randomly selected for whole-genome sequencing. Of the 4 sequenced strains, P10 and R46 were found to belong to ST113 [20], Lr12 belong to a new sequence type (ST5352) [21], and Lr2 to ST22. The isolate Lr2 was collected from blood culture in a hospital in Lahore, Pakistan, in March 2019. The isolate was preliminarily identified by biochemical tests (catalase, oxidase, and coagulase) [45]. The antimicrobial susceptibility was performed by disc diffusion method as per CLSI guidelines against the following antibiotics: ampicillin (10 µg), oxacillin (1 µg), methicillin (10 µg), vancomycin (5 µg), gentamicin (10 µg), erythromycin (15 µg), streptomycin (25 µg), clindamycin (2 µg), ciprofloxacin (5 µg), chloramphenicol (30 µg), linezolid (30 µg), tetracycline (30 µg), rifampicin (5 µg), and fusidic acid (10 µg) [46]. Furthermore, resistance to methicillin was confirmed by PCR amplification of the *mecA* gene [20].

### 4.2. Genome Sequencing, Assembly, and Annotation

The genomic DNA (gDNA) was extracted from a fresh culture of Lr2 by Invitrogen^®^ DNA extraction kit Cat no. K1820-01 (Thermo Fisher Scientific, Carlsbad, CA, USA). The gDNA integrity was checked by 0.7% agarose gel and quantified by Qubit 2.0 fluorometer Cat no. Q32866 (Manufactured by Tecan Austria GmbH, Grodig, Austria for Life Technologies). The gDNA libraries were prepared by Nextera XT Library Prep Kit (Illumina, San Diego, CA, USA), which was used as per instructions with a slight modification. Sequencing was performed in Illumina (HiSeq) system using a 250 bp paired-end protocol, and Trimmomatic 0.30 was used to trim adapters from raw reads [47]. The resultant reads were de novo assembled using SPAdes version 3.7 [48], and the generated contigs of Lr2 were annotated by NCBI’s Prokaryotic Genome Annotation Pipeline (PGAP) [49]. Finally, the assembled contigs were reordered with *S. aureus* reference genome NCTC8325 using Mauve [50], and the genome was visualized using CGView server (http://cgview.ca/, accessed on 3 December 2021) [51].

### 4.3. Genome-Based Characterization

In silico epidemiological characteristics of the assembled genome were carried out using SCCmecFinder-1.2 for the identification of *SCCmec* type [52], MLST 1.8 [53] for Multilocus Sequence Typing, and *spa*Typer 1.0 [54] for *spa* typing available at Center for Genomic Epidemiology (CGE) webserver (https://cge.cbs.dtu.dk/services/, accessed on 22 August 2021).

### 4.4. Prediction of Resistome, Virulome, and Mobilome

The chromosomal mutations and acquired genes conferring antibiotic resistance were identified by ResFinder 4.1 at CGE (https://cge.cbs.dtu.dk/services/ResFinder/, accessed on 22 August 2021) [55]. Virulence factors in the genome were identified and annotated using the Virulence Factor Database (VFDB) at http://www.mgc.ac.cn/VFs/, accessed on 22 August 2021 [56]. The assembled genome was searched for plasmid replicons (*rep*) in PlasmidFinder 2.1 using default parameters [57]. The identified plasmid replicons (*rep*) were searched in PLSDB (Plasmid Database) for the identification of full-length plasmids, and the full-length plasmids were then BLASTed with the Lr2 genome [58]. The prophage sequences in the assembled genome were identified and annotated by PHASTER online tool [59]. The identified plasmids and prophages were also investigated for genes associated with antimicrobial resistance and virulence.

### 4.5. Whole-Genome Single Nucleotide Polymorphism (SNP)-Based Phylogenetic Analysis 

The whole-genome SNPs phylogenetic analysis of MRSA Lr2 ST22 strain was analyzed against a set of publicly available ST22 complete genomes (*n* = 19, according to PATRIC, https://www.patricbrc.org/, accessed on 29 March 2021). The SNPs were called against the reference genome NCTC_8325 (GenBank accession no. CP000253.1), and the maximum-likelihood tree was established using FastTree 2 tool [60] in CSI Phylogeny (https://cge.cbs.dtu.dk/services/CSIPhylogeny/, accessed on 15 April 2021) [61]. The following default settings were applied: SNP positions minimum depth 10, SNP positions relative depth 10, a minimum distance between SNPs (prune) 10, minimum quality of SNP 30, and minimum Z-score of SNP 1.96. The phylogenetic tree was visualized, and a heatmap of the presence and absence of antibiotic resistance genes and virulence factors was generated using Interactive Tree of Life (iTOL) [62].

### 4.6. Pan-Genome and Cluster of Orthologous Genes (COGs) Analysis 

The MRSA ST22 genomes were annotated by Prokka at default parameters for estimation of pan genome [63]. The pangenome analysis and core-genome SNPs-based phylogenetic analysis were performed using the pangenome estimation module (PGM) of an in-house pipeline PanRV, which makes use of Roary [64,65]. The functional annotation of core genome of ST22 strains and COGs unique to Lr2 ST22 strain was performed using the functional annotation analysis module (FAM) of the in-house PanRV pipeline with 0.001 E-value, bit score 100, and percentage identity 70 [66,67].

## 5. Conclusions

This study provides important epidemiological and genomic data on a PVL-positive CA-MRSA ST22 strain Lr2 from Pakistan. The comparative analysis of the resistome shows that, unlike other ST22 strains, the strain Lr2 carries a plasmid-associated antibiotic resistance gene *erm(C)* and an aminoglycoside resistance gene *aac(6′)-Ie-aph(2″)-Ia*. The strain also harbors several prophages with genes encoding important *S. aureus* virulence factors such as PVL and TSST. These antibiotic resistance- and virulence-associated genes possibly make this ST22 clone highly antibiotic-resistant and virulent, and these genes could be transferred to other MRSA clones through horizontal gene transfer. However, further studies are needed on a large collection of isolates to determine the molecular epidemiology, evolution, and dynamics of transmission of this clone in Pakistan.

## Figures and Tables

**Table 1:** The phenotypic and genotypic resistance profile of Lr2.

Antibiotic	Sensitivity (Zone of Growth Inhibition in mm)	Related Gene(s)
Oxacillin	R (no zone)	*mecA*
Ampicillin	R (no zone)	
Methicillin	R (no zone)	
Gentamicin	R (4 mm)	*aac(6′)-Ie-aph(2″)-Ia*
Streptomycin	R (7 mm)	
Erythromycin	R (4 mm)	*erm(C)*
Clindamycin	R (9 mm)	
Ciprofloxacin	R (11 mm)	*gyrA*
Vancomycin	S (23 mm)	ND
Chloramphenicol	S (17 mm)	ND
Linezolid	R (13 mm)	ND
Rifampicin	S (21 mm)	ND
Tetracycline	R (9 mm)	ND
Fusidic acid	R (11 mm)	ND

R—resistant; S—susceptible; ND—not determined.

**Table 2:** The genomic features and characteristics of the MRSA strain Lr2 ST22.

Characteristics	Lr2
Genome size (bp)	2,831,239
Contigs	52
GC content %	32.7
N_50_	131,746
N_75_	94,139
L_50_	7
Largest contig (bp)	425,597
Genes (total)	2835
CDSs (total)	2768
No. of tRNA	56
No. of rRNA	4, 2, 1 (5S, 16S, 23S)
ST	22
SCC*mec* type	IVa(2B)
*spa*-type	t2986
NCBI Accession number	JAIGOF000000000

**Table 3:** Characteristics of prophages present in MRSA Lr2 ST22.

Prophage	Length (Kb)	Total Proteins	Phage Hit Proteins	GC %	Annotation	Most Common Phage	Virulence Factors
1 (incomplete)	9	15	7	32.8	Transposase, tail	PHAGE_Bacill_IEBH	None
2 (incomplete)	19	24	20	31.6	Integrase	PHAGE_Staphy_PT1028	*sec, sell, tsst*
3 (incomplete)	9.1	20	10	29.0	Head	PHAGE_Clostr_phiC2	None
4 (incomplete)	42.8	42	35	31.9	Integrase	PHAGE_Staphy_phiPVL_CN125	*ebp, lukF-PV, lukS-PV*
5 (incomplete)	12.5	24	21	28.8	Portal, transposase	PHAGE_Staphy_96	*seg, sei, yent2, selm, seln, selo*
6 (complete)	55.1	79	68	32.6	Tail, head, portal, terminase, integrase	PHAGE_Staphy_tp310_3	*sak, chp, scn*
7 (questionable)	46	37	26	33.2	Tail, transposase, integrase	PHAGE_Staphy_phiN315	*cna*

## Data Availability

The PVL-positive CA-MRSA strain Lr2 ST22 whole-genome sequence reads has been submitted in the NCBI SRA (SRR15497842) and sequence data in NCBI GenBank (JAIGOF000000000) under the BioProject PRJNA520768.
